# Case Report: Anti-N-methyl-D-aspartate receptor encephalitis associated with bilateral mature ovarian teratomas in early pregnancy

**DOI:** 10.3389/frph.2026.1895027

**Published:** 2026-07-22

**Authors:** Gulshat Bilyalova, Elmira Gassanova, Dinara Turzhanova, Saira Iskalieva, Aigerim Butabekova, Aisulu Tulemissova, Bahit Anapina, Assel Boshanova, Aida Baibusunova, Zhansulu Akylzhanova

**Affiliations:** 1 Department of Obstetrics and Gynecology No. 2, Astana Medical University, Astana, Kazakhstan; 2 Department of Obstetrics and Gynecology, Multidisciplinary City Hospital No. 2, Ministry of Health of the Republic of Kazakhstan, Astana, Kazakhstan; 3 Department of Obstetrics and Gynecology, Semey Medical University, Semey, Kazakhstan; 4 Department of Sports Education, Kazakh National University of Sports, Astana, Kazakhstan

**Keywords:** anti-NMDAR encephalitis, immunotherapy, MRI, ovarian teratoma, pregnancy

## Abstract

Anti-N-methyl-D-aspartate receptor (NMDAR) encephalitis is an autoimmune encephalitis that may present with acute psychiatric symptoms, seizures, movement disorders, impaired consciousness and autonomic dysfunction. A 30-year-old previously healthy woman at 10-11 weeks of gestation was admitted to the intensive care unit after an approximately 2-3-week history, reported by relatives, of insomnia, behavioral change, auditory and visual hallucinations, psychomotor agitation, aggressive behavior, and a generalized tonic-clonic seizure before admission. On admission, the patient had severe encephalopathy and subsequently required mechanical ventilation because of hypoventilation. Cerebrospinal fluid testing detected IgG antibodies against NMDAR at a titer of 1:160. Brain magnetic resonance imaging showed a right hippocampal lesion consistent with limbic encephalitis, and pelvic ultrasonography identified adnexal masses suspicious for teratoma. Prolonged EEG demonstrated diffuse delta-theta slowing with a delta-brush pattern, without findings consistent with focal or myoclonic seizures. The patient received high-dose methylprednisolone, intravenous immunoglobulin, plasma exchange, rituximab, pregnancy-conscious antiseizure therapy, individualized pregnancy management, and laparoscopic removal of bilateral mature ovarian teratomas. Following combined immunotherapy, ventilatory and supportive ICU care, antiseizure therapy, individualized maternal-fetal management, and tumor removal, the recurrent motor events ceased by the end of the first postoperative week. The patient's consciousness, speech, and motor function gradually improved. This case highlights that anti-NMDAR encephalitis in pregnancy requires early cerebrospinal fluid antibody testing, exclusion of infectious mimics, supportive neurocritical care, prompt immunotherapy, timely teratoma removal and individualized reasoning regarding pregnancy management.

## Introduction

Anti-N-methyl-D-aspartate receptor (NMDAR) encephalitis is an antibody-mediated autoimmune encephalitis characterized by IgG antibodies directed against the GluN1 subunit of the NMDAR (1, 2). The syndrome frequently evolves over days to weeks, often beginning with psychiatric symptoms before seizures, speech disturbance, dyskinesias, impaired consciousness, or autonomic instability (1). Because early manifestations may resemble primary psychiatric disease, infectious encephalitis, toxic-metabolic delirium, medication-related delirium or other autoimmune encephalitides, diagnosis can be delayed unless autoimmune encephalitis is considered early.

Young women are disproportionately affected, and ovarian teratomas are among the most important associated tumors (2, 3). Teratomatous neural tissue may express NMDAR-related antigens, providing a plausible source for intrathecal antibody production (3). For this reason, tumor screening and timely tumor removal are considered disease-directed interventions in addition to immunotherapy (4).

Anti-NMDAR encephalitis during pregnancy is rare and requires close collaboration among neurologists, obstetricians, gynecologists, intensivists, infectious disease specialists, anesthesiologists, psychiatrists, neonatologists, and rehabilitation teams (5–11). We report a severe first-trimester case at 10–11 weeks of gestation associated with bilateral mature ovarian teratomas, hypoventilation requiring mechanical ventilation, and individualized pregnancy management.

## Case description

A 30-year-old woman (G2P1A0) with no relevant prior neurologic, psychiatric, autoimmune, or oncologic history was brought to the emergency department because of acute neuropsychiatric deterioration (Table 1). According to relatives, symptoms had been evolving for approximately 2 weeks before admission, beginning with insomnia and followed by progressive behavioral change, auditory and visual hallucinations, psychomotor agitation, aggressive behavior, and impaired orientation. A generalized tonic-clonic seizure occurred before admission. No relevant family history was reported.

**Table 1:** Timeline of key clinical events, diagnostic findings, treatment, and follow-up.

Time point	Event
Several days before admission	The patient developed insomnia followed by progressive behavioral change, auditory and visual hallucinations, psychomotor agitation, aggressive behavior and impaired orientation.
Illness day 7/admission day 0	A generalized tonic-clonic seizure occurred before admission. The patient was admitted to the ICU with a Glasgow Coma Scale score of 10–11, blurred consciousness and slow reaction.
Hospital days 0–3	Infectious evaluation was negative. Brain MRI showed hyperintense signal in the right hippocampal region. Prolonged EEG showed diffuse delta-theta slow wave activity with a delta-brush pattern. CSF NMDAR-IgG was reported positive by day 3. LGI1, CASPR2, and GAD antibodies were negative. Pelvic ultrasonography showed mixed-echogenicity ovarian masses.
Hospital days 1–5	First-line immunotherapy and antiseizure treatment was initiated on hospital day 1 after exclusion of key infectious mimics. During ICU, recurrent myoclonus-like events remained after first-line treatment. No generalized tonic-clonic seizures were reported in the ICU. The patient's condition improved slightly, but cognitive impairment persisted.
Hospital days 5–10	Therapeutic PLEX was performed for 3 sessions, and rituximab was administered. Pregnancy termination by vacuum aspiration was performed.
Hospital day 10	Laparoscopic removal of bilateral ovarian teratomas was performed.
Postoperative days 1–7	No seizures were reported and the recurrent myoclonus-like events ceased by the end of the first postoperative week.
Discharge	The patient had clear consciousness and recovered phrase-level speech, with mild residual emotional lability and distal limb weakness. She was transferred to a neurologic rehabilitation unit.
3-month follow-up	Mental status had nearly normalized, although memory impairment persisted.

On admission to the intensive care unit, the patient's condition was severe, with a Glasgow Coma Scale score of 10–11. Neurologic examination revealed blurred consciousness, restlessness, slow reaction to external stimuli, poor memory and impaired orientation. The Barthel Index score was 55 points, indicating substantial functional dependence. During the ICU course, the patient developed hypoventilation requiring mechanical ventilation. Initial laboratory testing showed moderate anemia with hemoglobin 8.5 g/dL and leukopenia. Admission routine tests were not suggestive of alternative systemic, endocrine or malignant disease.

### Diagnostic assessment

The working diagnosis was anti-NMDAR encephalitis associated with ovarian teratomas. Infectious encephalitis, primary psychiatric disease, structural brain disease and other encephalitides were considered in the differential diagnosis and were evaluated with virologic studies, CSF antibody testing, neuroimaging and tumor screening.

Serologic studies for cytomegalovirus, herpes simplex virus, Epstein–Barr virus and other viral infections were negative. CSF analysis showed mild pleocytosis, with normal protein, glucose, and chloride levels. Neuronal antibody testing by immunofluorescence detected IgG antibodies against NMDAR in CSF at a titer of 1:160. This result was reported by day 3 and confirmed the working diagnosis. Antibodies against leucine-rich glioma-inactivated 1, contactin-associated protein-like 2 and glutamic acid decarboxylase were negative. Repeat CSF or serum NMDAR-IgG testing was not performed during hospitalization. Therefore, the duration of antibody positivity could not be assessed.

Brain CT showed no evidence of acute ischemia or intracranial hemorrhage. Brain MRI demonstrated hyperintense signal in the right hippocampal area, consistent with limbic encephalitis. Prolonged EEG showed diffuse delta-theta slow-wave activity with a delta-brush pattern. Monitoring showed no electrographic seizures, status epilepticus, or findings consistent with focal or myoclonic seizures.

Gynecologic assessment confirmed an intrauterine pregnancy at 10–11 weeks of gestation. Abdominal and pelvic ultrasonography revealed a mixed-echogenicity left ovarian mass measuring 7 × 8 cm, compatible with teratoma. Marker assessment showed beta-hCG 142,000 mIU/mL, AFP (21.5 ng/mL) and LDH (92 U/L) within the normal range. CA-125 and HE4 levels were not suggestive of epithelial ovarian malignancy.

### Therapeutic intervention

Management was performed by a multidisciplinary team including neurology, obstetrics and gynecology, intensive care, anesthesiology, infectious disease, psychiatry, and rehabilitation specialists. ICU admission was required because of impaired consciousness, agitation, recurrent seizure risk and central hypoventilation requiring mechanical ventilation. Supportive ICU care included serial GCS assessment, cardiopulmonary and hemodynamic monitoring, ventilatory support, seizure and aspiration precautions, fluid and nutritional support.

Immunotherapy was initiated on hospital day 1 with intravenous pulse methylprednisolone 1,000 mg/day for 5 days and intravenous immunoglobulin 0.4 g/kg/day for 5 days. This is consistent with contemporary autoimmune encephalitis recommendations, which advise that empiric treatment should not be delayed when clinical suspicion is high and infectious mimics have been reasonably excluded (12, 13). After first-line immunotherapy, the mental state improved slightly, but obvious cognitive impairment and recurrent clinically observed myoclonus-like events persisted, supporting escalation and continued tumor-directed management. Therapeutic plasma exchange was performed for 3 sessions, and the first dose of rituximab 1,000 mg was administered as second-line immunotherapy. Escalation to PLEX and rituximab was not considered an indication for pregnancy termination because these interventions have been reported as feasible in selected pregnant patients when maternal disease severity warrants treatment (5, 6).

Antiseizure treatment was initiated in the ICU. Levetiracetam was selected as the primary antiseizure medication because of its comparatively favorable pregnancy safety profile and was administered at 1,000 mg daily, with planned titration by 500 mg twice daily at approximately two-week intervals if recurrent seizures occurred, up to 1,500 mg twice daily. Lamotrigine was added as adjunctive maintenance therapy at 25 mg once daily for two weeks, followed by 50 mg once daily for two weeks. This selection was consistent with antiseizure medication guidance favoring lower-risk options when feasible and avoiding valproate and topiramate in patients who are pregnant or may become pregnant because of fetal and neurodevelopmental risks (14, 15).

One generalized tonic-clonic seizure occurred before admission, and no generalized tonic-clonic seizures were reported in the ICU. Recurrent myoclonus-like events occurred after initiation of first-line immunotherapy and persisted until hospital day 10, with approximately 1–2 events daily.

Pregnancy termination was not considered routine treatment for anti-NMDAR encephalitis and was not pursued as a substitute for tumor removal. The pregnancy-management decision was made only after individualized multidisciplinary risk assessment. The patient-specific concerns were severe deterioration, central hypoventilation requiring mechanical ventilation, recurrent myoclonus-like motor events, and insufficient clinical improvement after initial immunotherapy. Because the patient's ability to provide informed consent was limited at that time, the decision-making process involved discussion with a family representative according to institutional practice and shared decision-making principles in obstetric care (16).

On hospital day 10, laparoscopic surgery for removal of the ovarian tumors was performed as the disease-directed intervention aimed at removing the presumed antigenic source. Intraoperatively, the right ovary contained a multilocular spherical mass measuring 7.5 × 8.0 cm, and the right fallopian tube appeared grossly normal. On the left side, the ovary was cystically enlarged with an irregular spherical contour. A dermoid cyst with a dense capsule measuring 5.0 × 6.0 cm was identified. Histopathologic examination confirmed bilateral mature ovarian teratomas containing adipose tissue, hair follicles, sebaceous glands, glial tissue, cartilage, and bone.

### Follow-up and outcomes

During the postoperative period, the patient showed gradual neurologic improvement. Following combined immunotherapy, ventilatory and supportive ICU care, antiseizure therapy, individualized maternal-fetal management, and laparoscopic removal of bilateral ovarian teratomas, the recurrent motor events ceased by the end of the first postoperative week.

At discharge, the patient had clear consciousness and recovered speech to spontaneous phrase-level production. Mild residual emotional lability and distal limb weakness remained. The patient was transferred to a neurologic rehabilitation unit for further recovery.

At 3-month follow-up, the patient's mental status had nearly normalized, and no seizures were reported. Residual memory impairment persisted, indicating incomplete cognitive recovery.

## Discussion

Current diagnostic and acute-management guidance of anti-NMDAR encephalitis emphasizes syndromic recognition, exclusion of infectious and toxic-metabolic mimics, MRI/EEG/CSF assessment, tumor screening, and initiation of empiric immunotherapy when suspicion is high rather than waiting for antibody confirmation (1, 12, 13). CSF antibody testing is particularly important because serum-only NMDAR-IgG testing may be less sensitive and can be more difficult to interpret (1).

Pregnant patients usually present with the typical syndrome of anti-NMDAR encephalitis rather than a pregnancy-specific phenotype: acute psychiatric or behavioral change, hallucinations, seizures, dyskinesias or abnormal movements, impaired consciousness, autonomic dysfunction and central hypoventilation (5–7, 9, 10). In the 2020 series by Joubert et al., 7 of 11 collected patients (64%) and 18 of 21 previously reported patients (86%) required ICU admission. Seizures were frequent, and central hypoventilation occurred in 3 of 11 patients (27%) in the new series and 9 of 21 patients (43%) in the reviewed literature cases (5). In the earlier review by Shi et al., all patients initially presented with psychiatric symptoms, 8 of 13 cases began in the first trimester, and hypoventilation severe enough to require intubation, tracheotomy or mechanical ventilation was reported in 7 of 13 patients (9).

The presence of hypoventilation is clinically important in pregnancy because it reflects severe brainstem/autonomic involvement and creates direct maternal-fetal risk through hypoxemia, aspiration risk, hemodynamic instability, need for sedatives and prolonged intensive monitoring. Crowley et al. described a similar early-pregnancy presentation in which a patient developed erratic behavior, hallucinations, a prolonged generalized tonic-clonic seizure, reduced consciousness, and central hypoventilation requiring intubation and transfer for higher-level care (6).

Tumor assessment requires both imaging and marker interpretation. Mature teratoma was the leading preoperative diagnosis, but mixed germ-cell tumor components must be considered because yolk sac tumor may increase AFP, dysgerminoma may increase LDH, and choriocarcinoma or trophoblastic components may increase beta-hCG (17). In this report, first-trimester beta-hCG was interpreted in the context of pregnancy, while AFP and LDH were not suggestive of malignant or mixed germ-cell components. Histopathology confirmed bilateral mature ovarian teratomas containing glial tissue and other mature elements. This finding is central to the pathophysiologic interpretation of the case because teratomatous neural tissue may express NMDAR-related antigens and provide an antigenic stimulus for B-cell activation and intrathecal antibody production (3).

Surgical series and anti-NMDAR encephalitis literature support timely tumor removal when feasible, and published pregnancy cases also emphasize identification and resection of ovarian teratoma when present (4, 6, 7, 10). In the present case, cessation of recurrent motor events within the first postoperative week was temporally consistent with the combined effect of immunotherapy, supportive care, antiseizure management, and tumor-directed treatment.

Early treatment is a major prognostic principle. In the 577-patient cohort by Titulaer et al., 81% of patients had a good outcome at 24 months, early treatment was associated with better outcomes, and second-line immunotherapy was associated with better outcomes among patients who did not improve after first-line therapy (2). Accordingly, patient received high-dose methylprednisolone and IVIG promptly, followed by PLEX and rituximab because severe cognitive impairment and recurrent motor events persisted. This escalation is consistent with contemporary autoimmune encephalitis recommendations and with the treatment pattern described in pregnancy-specific literature (5, 6, 9, 12, 13).

In Joubert et al., immunotherapy was used during pregnancy in 7 of 11 patients from the authors' cohort and in 18 of 21 previously reported cases. Treatment included corticosteroids, IVIG, plasma exchange, and rituximab, and rituximab was used in four pregnant patients without reported adverse effects in mothers or infants (5). Crowley et al. also stated that high-dose corticosteroids, IVIG, and plasma exchange are used in pregnancy, while rituximab may be appropriate in treatment-refractory cases, with relatively low placental transfer during the first trimester (6).

Anti-NMDAR encephalitis may cause generalized tonic-clonic seizures, focal or nonconvulsive seizures, status epilepticus, and dyskinetic or myoclonus-like movements that can be difficult to distinguish clinically. In this case, prolonged EEG did not show electrographic seizures despite recurrent observed motor events, supporting continued clinical-EEG correlation. Levetiracetam and lamotrigine were reasonable pregnancy-conscious choices, consistent with guidance favoring lower-risk antiseizure medications when feasible and avoiding valproate and topiramate during pregnancy because of fetal and neurodevelopmental risks (14, 15).

The possible relationship between pregnancy and anti-NMDAR encephalitis remains unresolved. Shi et al. proposed several mechanisms by which pregnancy might influence disease expression, including pregnancy-related immune modulation, hormone effects on autoreactive B and T cells, possible embryo or placental antigenic stimulation, and the observation that NMDAR expression has been identified in ovum (9). These hypotheses are biologically plausible because pregnancy is characterized by major endocrine and immunologic changes, because anti-NMDAR encephalitis disproportionately affects women of reproductive age, and because many published pregnancy-associated cases began in early gestation. Current literature does not prove that pregnancy itself is a sufficient cause of anti-NMDAR encephalitis, and it does not show that pregnancy termination reliably stops disease progression (5, 7, 9–11). Thus, pregnancy may represent a permissive or modifying immunological context in some patients.

In the present case, pregnancy itself was considered as a possible trigger or immune-modifying context, but the bilateral mature ovarian teratomas were considered the central and more convincing disease driver. First, ovarian teratoma is a well-established antigenic source in anti-NMDAR encephalitis, whereas pregnancy as an independent causal trigger remains uncertain. Second, the available literature includes favorable maternal recovery and healthy neonatal outcomes after pregnancy continuation when immunotherapy, ICU support, and tumor management are individualized (5–7, 11). Kumar et al. reported three pregnant patients with anti-NMDAR encephalitis: symptoms began at 14, 8, and 17 weeks of gestation, two patients had ovarian teratomas, all had substantial neurologic recovery, and the two newborns were healthy (7). In the single patient whose pregnancy was terminated, the authors attributed the decision to severe neurologic symptoms, recurrent bilateral teratomas and early gestational stage (7).

Chan et al. provide an additional reason not to overstate the therapeutic role of pregnancy loss. In their case, miscarriage occurred within two days of hospitalization, but the patient underwent teratoma removal and required more than four months of intensive care before gradual recovery (10). This case suggests that pregnancy loss does not necessarily halt the encephalitic process.

The pregnancy-specific literature therefore supports individualized multidisciplinary management rather than a single algorithm. Crowley et al. emphasized multidisciplinary care for critically ill obstetric patients, including neurology, anesthesiology, obstetrics, perinatal mental health, ICU care, regular team meetings, timing of surgery, immunotherapy decisions, psychosis management, delivery planning, and rehabilitation (6).

Several limitations should be acknowledged. The case is a single observation, and clinical improvement followed multiple interventions, including high-dose corticosteroids, IVIG, plasma exchange, rituximab, antiseizure therapy, mechanical ventilation and supportive ICU care, pregnancy management, and bilateral teratoma removal. Repeat serum or CSF NMDAR-IgG titers were not obtained, so antibody kinetics could not be compared with the timing of surgery or neurologic recovery.

## Conclusion

Anti-NMDAR encephalitis should be considered in pregnant patients with acute psychiatric symptoms, seizures, dyskinesias, autonomic dysfunction, impaired consciousness, hypoventilation, particularly when an ovarian teratoma is detected. This case highlights the need for early CSF antibody testing, exclusion of infectious mimics, prompt immunotherapy, neurocritical supportive care including mechanical ventilation when central hypoventilation occurs, pregnancy-conscious antiseizure treatment, and timely teratoma removal.

## Patient perspective

The hospitalization was a difficult period for me and my family because the disease required urgent decisions about my treatment. After treatment, my daily functioning gradually improved. Although I still experience some difficulties with reading, I can say that my condition is much better than it was during hospital stay.

## Data Availability

The original contributions presented in the study are included in the article/Supplementary Material, further inquiries can be directed to the corresponding author.

## References

[1] Graus F Titulaer MJ Balu R Benseler S Bien CG Cellucci T. A clinical approach to diagnosis of autoimmune encephalitis. Lancet Neurol. (2016) 15(4):391–404. 10.1016/S1474-4422(15)00401-9 26906964 PMC5066574

[2] Titulaer MJ McCracken L Gabilondo I Armangué T Glaser C Iizuka T. Treatment and prognostic factors for long-term outcome in patients with anti-NMDA receptor encephalitis: an observational cohort study. Lancet Neurol. (2013) 12(2):157–65. 10.1016/S1474-4422(12)70310-1 23290630 PMC3563251

[3] Wu CY Wu JD Chen CC. The association of ovarian teratoma and anti-N-methyl-D-aspartate receptor encephalitis: an updated integrative review. Int J Mol Sci. (2021) 22(20):10911. 10.3390/ijms222010911 34681570 PMC8535897

[4] Dai Y Zhang J Ren H Zhou X Chen J Cui L. Surgical outcomes in patients with anti-N-methyl-D-aspartate receptor encephalitis with ovarian teratoma. Am J Obstet Gynecol. (2019) 221(5):485.e1–485.e10. 10.1016/j.ajog.2019.05.026 31128109

[5] Joubert B García-Serra A Planagumà J Martínez-Hernandez E Kraft A Palm F. Pregnancy outcomes in anti-NMDA receptor encephalitis: case series. Neurol Neuroimmunol Neuroinflamm. (2020) 7(3):e668. 10.1212/NXI.0000000000000668 31948997 PMC7051205

[6] Crowley CM Liew NC Plans C O'Brien S Imcha M. Successful multi-disciplinary management of anti-NMDAR encephalitis during pregnancy. Obstet Med. (2025) 18(1):33–5. 10.1177/1753495X231190594 39959000 PMC11826843

[7] Kumar MA Jain A Dechant VE Saito T Rafael T Aizawa H. Anti-N-methyl-D-aspartate receptor encephalitis during pregnancy. Arch Neurol. (2010) 67(7):884–7. 10.1001/archneurol.2010.133 20625099

[8] Kim J Park SH Jung YR Park SW Jung DS. Anti-NMDA receptor encephalitis in a pregnant woman. J Epilepsy Res. (2015) 5(1):29–32. 10.14581/jer.15008 26157673 PMC4494994

[9] Shi YC Chen XJ Zhang HM Wang Z Du DY. Anti-N-methyl-D-aspartate receptor (NMDAR) encephalitis during pregnancy: clinical analysis of reported cases. Taiwan J Obstet Gynecol. (2017) 56(3):315–9. 10.1016/j.tjog.2017.04.009 28600040

[10] Chan LW Nilsson C Schepel J Lynch C. A rare case of anti-N-methyl-D-aspartate receptor encephalitis during pregnancy. N Z Med J. (2015) 128(1411):89–91. 25820508

[11] Kokubun N Komagamine T Hirata K. Pregnancy and delivery in anti-NMDA receptor encephalitis survivors. Neurol Clin Pract. (2016) 6(5):e40–3. 10.1212/CPJ.0000000000000229 29443276 PMC5765914

[12] Abboud H Probasco JC Irani S Ances B Benavides DR Bradshaw M. Autoimmune encephalitis: proposed best practice recommendations for diagnosis and acute management. J Neurol Neurosurg Psychiatry. (2021) 92(7):757–68. 10.1136/jnnp-2020-325300 33649022 PMC8223680

[13] Hahn C Budhram A Alikhani K AlOhaly N Beecher G Blevins G. Canadian Consensus guidelines for the diagnosis and treatment of autoimmune encephalitis in adults. Can J Neurol Sci. (2024) 51(6):734–54. 10.1017/cjn.2024.16 38312020

[14] Pack AM Oskoui M Williams Roberson S Donley DK French J Gerard EE. Teratogenesis, perinatal, and neurodevelopmental outcomes after in utero exposure to antiseizure medication: practice guideline from the AAN, AES, and SMFM. Neurology. (2024) 102(11):e209279. 10.1212/WNL.0000000000209279 38748979 PMC11175651

[15] National Institute for Health and Care Excellence. Epilepsies in children, young people and adults. NICE guideline NG217. Published 27 April 2022; Accessed 30 January 2025).

[16] American College of Obstetricians and Gynecologists. Informed consent and shared decision making in obstetrics and gynecology. Committee opinion No. 819. Obstet Gynecol. (2021) 137:e34–41. 10.1097/AOG.0000000000004247 33481530

[17] Reed N Millan D Verheijen RHM Castiglione M; ESMO Guidelines Working Group. Non-epithelial ovarian cancer: eSMO clinical practice guidelines for diagnosis, treatment and follow-up. Ann Oncol. 2010;21 Suppl 5:v31–6. 10.1093/annonc/mdq205 20555098

